# Association of biomass fuel use with acute respiratory infections among under- five children in a slum urban of Addis Ababa, Ethiopia

**DOI:** 10.1186/1471-2458-14-1122

**Published:** 2014-10-31

**Authors:** Habtamu Sanbata, Araya Asfaw, Abera Kumie

**Affiliations:** Department of Public and Environmental Health, College of Medicine and Health Science, Hawassa University, Hawassa, Ethiopia; Department of Physics and Center for Environmental Sciences, College of Natural Sciences, Addis Ababa University, Addis Ababa, Ethiopia; School of Public Health, College of Health Sciences, Addis Ababa University, Addis Ababa, Ethiopia

**Keywords:** Biomass fuel, Acute respiratory infection, Children, Slum, Addis Ababa, Ethiopia

## Abstract

**Background:**

Indoor air pollution from biomass fuel is responsible for 50,320 annual deaths of children under-five year, accounting for 4.9% of the national burden of disease in Ethiopia. Acute respiratory infections are the leading cause of mortality among children in Ethiopia. There is limited research that has examined the association between the use of biomass fuel and acute respiratory infections among children.

**Methods:**

A community based cross-sectional study was conducted during January to February 2012 among 422 households in the slum of Addis Ababa. Data were collected by using structured and pretested questionnaire. Odds ratio was done to determine association between independent variables and acute respiratory infections by using logistic regression analysis. Multivariate logistic regression was used to determine the presence of an association between biomass fuel use and acute respiratory infections after controlling for other confounding variables.

**Results:**

Nearly 253 (60%) of children live in households that predominately used biomass fuel. The two weeks prevalence of acute respiratory infection was 23.9%. The odds ratios of acute respiratory infection were 2.97 (95% CI: 1.38-3.87) and 1.96 (95% CI: 0.78-4.89) in households using biomass fuel and kerosene, respectively, relative to cleaner fuels.

**Conclusion:**

There is an association between biomass fuel usage and acute respiratory infection in children. The relationship needs investigation which measure indoor air pollution and clinical measures of acute respiratory infection.

## Background

Household energy affects health of the poor through a variety of physical, social and economic aspects. The most important direct health effect results from indoor air pollution (IAP) produced by burning biomass fuels and coal in simple stoves with inadequate ventilation [1]. Worldwide, more than three billion people depend on solid fuels, including biomass (wood, dung, crop residues, coal) for cooking and heating [2]. IAP is responsible for more than 1.6 million deaths annually and 2.7% of the global burden of disease. Acute respiratory infections (ARI) are the biggest killer of young children [1].

Biomass fuels are at the low end of the energy ladder in terms of combustion efficiency and cleanliness. Biomass combustion produces a large number of health-damaging air pollutants including respirable particulate matter, carbon monoxide (CO), nitrogen oxides, formaldehyde, benzene, 1,3 butadiene, polycyclic aromatic hydrocarbons(PAHs)and many other toxic organic compounds [3].

Over 70 percent of energy needs in most of the sub-Saharan African countries are met by biomass fuels, mainly for the household. The efficiency of fuel wood used for cooking in developing countries is quite low, leading to energy requirements that are several times higher than in developed countries [4]. Developing countries are significantly affected by the problem due to their increased use of polluting energy sources for their energy demands. World Health Organization (WHO) estimates on burden of diseases for the year 2002, IAP from biomass use accounted for 3.7% of the burden of disease in developing countries [1].

In Ethiopia, about 95% of the country’s energy supply comes from biomass sources [5]. IAP from biomass fuel is responsible for 50,320 annual ARI deaths of children under-five year, accounting 4.9% of national burden of disease in Ethiopia [1]. The estimated energy consumption per household in Addis Ababa is approximately 7 GJ, slightly less than half the per capita consumption. Traditional fuels (fuel wood, charcoal and dung) meet about 75% of household energy needs. Kerosene, Liquefied Petroleum Gas (LPG) and electricity provide the remaining 25%. In the last decade, kerosene use in Addis Ababa has declined considerably as a result of a doubling in the cost of kerosene, largely because of the removal of subsidies. At the same time, the price of electricity has declined by more than 50% and it is currently sold at approximately 75% less than the price of kerosene [6]. A large proportion of kerosene users have shifted to fuel wood [7].

Recent study conducted in Addis Ababa indicated the average fine particulate matter (PM_2.5_) concentration measured in homes using biomass fuel far exceeded WHO guidelines [7]. The city highest 24-hour geometric mean of PM_2.5_ concentrations observed in households using predominantly biomass fuel , kerosene, and clean fuel are 1,134 μg/m^3^ (SD = 3.36), 637 μg/m^3^ (SD = 4.44), and 335 μg/m^3^ (SD = 2.51) respectively [7]. Thus, the IAP in the city is high. Biomass fuel will continue to be used by a large number of households for the foreseeable future. The burden of disease due to IAP is exceedingly affecting children in poor slum urban households in Addis Ababa. There is limited evidence that link biomass fuel use with ARI among under-five children in Ethiopia. The aim of this study was to assess the association of biomass fuel and ARI among children under-five in the urban slum of Addis Ababa.

## Methods

### Study design and setting

A community based quantitative cross- sectional study was conducted to answer the research objectives. Data were collected using structured questionnaire by administering face to face interviewing of mothers. The study was conducted in Addis Ababa, the capital city of Ethiopia. The city is situated at the center of the country at an altitude varying between 2,200 and 2,800 meters above sea level (masl), between latitude 9.0300° N and longitude 38.7400° E. Average annual temperatures range from 8.2°C to 25.1°C. The administrative structure of the city Addis Ababa has 10 sub-cities and 116 districts (*woreda*s). The city has a projected population of approximately 3.4 million people and over 500,000 households with an average family size of six. With an estimated area of 530.14 square kilometers, this chartered city has an estimated density of 5,165.1 inhabitants per square kilometer [8]. An estimated 80% of the population in Addis Ababa lives in poor and overcrowded districts [9]. The study unit embraced all households with under-five children. Sampling units were households with children under five years in Addis Ababa.

### Sample size and sampling procedure

The sample size was calculated using a single population proportion formula using the following parameters: 95% confidence level (1.96), margin of error (0.05), expected prevalence of children with acute respiratory infection 50%. With this assumption a total of 422 household with children under- five were included in this study. The sampling procedure involved a three-staged sampling scheme aimed at obtaining 106 households in each selected sub-city using a probability to population size. A sub-city is the largest administrative structure in the city. In the first stage, total ten sub-cities were stratified into four based on similarity of crowdedness, housing situation and sanitation coverage of the city. From each strata one sub-city was selected by lottery method and thus four total sub-cities were selected from ten of the city. In the second stage, each Districts (*Woredas*) was clustered and three districts *(woredas*) from each of the four cities (total 12 districts) were selected targeting 34 to 37 households per district *(woreda).* In the third stage selection of houses having under-five children was conducted by using systematic random sampling method. This process have been then followed by a complete household listing operation, which was carried out in all the selected district *(woreda)* to provide a sampling frame for the third -stage selection of households with children under- five years.

### Data collection method

Data were collected during January to February 2012 through structured questionnaire through interviewing mothers of under- five children. The questionnaire was developed after critical discussions with public health experts. The questionnaire was adapted from WHO guidelines for survey of household fuel use [10] and for ARI module [11].

Data collectors were community health extension workers with similar professional experience. The data collectors were extensively trained by the principal investigator and a pediatrician for two days on interviewing techniques, observational, data recording, approaches to promote health education, and advocacy of child health screening. One professional expert was assigned in each sub-city for monitoring and supervision of data collection.

### Predictor variables

Information on types of fuel used for cooking was gathered. To elicit this information the question “What type of fuel does your household mainly use for cooking?” was asked. The question was then followed by a list of different fuel names to choose from. The choice include fuel wood, crop residues, dung cakes, charcoal, kerosene, Liquefied Petroleum Gas (LPG) and electricity. Exposure assessment questions also collected on socio demographic like sex of household head, family size, education level, cigarettes smoking habit, housing characteristics, type of stove used and ventilation efficiency, behavioral measures, most commonly whether child is carried by the mother while she is cooking also time spent near fire, and child stays in smoke, questions on location of the child relative to cooking place, cooking done in same room as where children sleeping were included. Use of biomass fuels kerosene, LPG and electricity is the main independent variable of the study.

### Child health screening

Questionnaire respondents were residents who voluntarily agreed to participate upon having the purpose of the research explained. Mothers were asked to describe the health status of children under five year within the household with respect to ARI. First, mothers were asked whether there were any children who were coughing. If the answer was yes, mothers were asked to describe the breathing (i.e.: short and/or rapid breathing). Children who suffered from coughs, accompanied by chest in drawing, shortness of breath, and rapid breathing were diagnosed with an acute respiratory infection, according to the WHO clinical case definition [11]. Suffering from ARI was the dependent variable used in this study.

### Operational definition

#### Acute respiratory infection (ARI)

This study uses the WHO definition, which defines ARI to include any combination of the following symptoms: cough with or without fever, blocked or runny nose, sore throat, and/or ear discharge with infection of the lungs. Serious forms of ARI result in pneumonia or bronchopneumonia [11]. Therefore, acute respiratory infections refer to upper respiratory and lower respiratory infections.

#### Acute lower respiratory infection

Serious form of ARI, resulting in pneumonia or bronchopneumonia.

#### Acute upper respiratory infection

Infection of the upper respiratory tract involving larynx, pharynx, tonsillar glands, Eustachian tube, nasal cavities and sinuses.

#### Fuel type

Fuel type refers to the source of energy used for cooking and was classified into three categories: biomass fuels (wood, dung cakes, charcoal, urban residues), kerosene, and clean fuel (LPG and electricity).

#### Improved cook stove

There is no international definition for the exact fuel savings that are necessary for a stove to be considered as an improved stove. However, an improved cook stove is a device that is designed to consume less fuel, save time, reduce the volume of smoke produced compared to the traditional stove.

#### Traditional cook stove

There is no universally accepted definition for traditional cook stoves that is linked to performance or technical standards. Thus, throughout this paper, the term refers to cook stoves constructed by household members that are not energy efficient and have poor combustion features using for cooking purpose.

#### Clean stove

Refers to efficient, safe, durable, and affordable stoves that use clean fuels. It includes both electric and LPG stoves.

#### Indoor air pollution

Indoor air pollution refers to the toxic emissions from cooking, especially from the use of wood, dung, charcoal, kerosene and other cooking fuels.

#### Cigarette smoking

Refers to any member of the household that smokes more than one cigarette a day at home.

#### Ventilation

Refers to the process of supplying or removing air from any enclosed space by natural or mechanical means. In this study, ventilation is measured by households that have properly built doors, windows (fanlight, hopper, or sliding sash windows), or mechanical apparatuses (fans and air conditioner).

### Data quality assurance

The questionnaire was written in English and administered in Amharic, the local language, by linguistic professionals. It was validated by independent repeat administration on consecutive days in approximately 10% of the households.

The pre-test was conducted among 5% of the respondents to test the efficacy of the research instruments and to discover possible weaknesses, inadequacies, and ambiguities so that they was able to corrected before actual data collection started. Data collection was overseen by a field supervisor who cross-check all field forms after each day of data collection to ensure that the forms were completely filled and data were consistent. Any mistake or omission was corrected as on the same day of data collection. Double data entry was used to check any inconsistency in responses from respondents.

### Data management and analysis

Coded data entry was done in EPI INFO version 6.04 for data clearance and skip pattern prior to analysis using the SPSS (Statistical Package of Social Science) Version 20.0 package. Cleaning was made to avoid missing values, outliers and other inconsistencies. Consistency and completeness of data were checked using frequency and a 2 by 2 tables. Descriptive statistics such as frequency distributions and measure of central tendencies were calculated for dependent and independent variables. Bivariate and multivariate analysis using binary logistic regressions were done to determine the presence of a statistically significant association between explanatory variables and the outcome variables. Crude Odds Ratio (OR) with 95% Confidence Intervals (CI) was done to determined association between independent variables and ARI. The multivariate logistic regression analysis was done using enter method hierarchically to assess the relative effect of the explanatory factors on acute respiratory infection. Multivariate logistic regressions analyses were used to determine the presence of an association between explanatory variables and acute respiratory infections by controlling the effect of other variables. To limit many variables and unstable estimates in the subsequent models, only variables that reached a p-value less than 0.30 at the bivariate analysis level were kept in the model. Data were presented using figures and tables.

### Ethical consideration

The study had ethical approval from Addis Ababa University and Health office of Addis Ababa. The study was in words communicated to each respondent and verbal consent was secured once agreement on the interview was obtained. Inconveniences for refusals were respected. Children whom found in their households with severe pneumonia were referred for treatment to near health institution. Results of the study will be planned to be communicated to the local authorities and community.

## Results

### Socio-demographic characteristics

A total of 422 samples in Addis Ababa households were involved in this study. The overall average family size was 5 persons per household. There were 285 (67.5%) male head of households and 137 (32.5%) were female-headed. The marital status distribution of house hold head were344 (81.5%) married, 31 (7.3%) single, 26 (6.2%) divorced, and 21 (5.0%) were widowed. The religion of the households head were predominantly Orthodox Christian and Muslim, 264 (62.6%) and 106 (25.1%) respectively, the remaining 52 (12.3%) were Protestant.

In terms of smoking habit, 62 (14.7%) had at least one member within the household who smokes more than one cigarette a day at home compared with 360 (85.3%) non-smokers (see Table 1).

**Table 1:** **Socio-demographic characteristic of sampled households in Addis Ababa, February 2012**

Socio demographics		Frequency	Percent
**Response rate**		422	100
**Sex of the household head**	Male	285	67.5
	Female	137	32.5
**Family size**	Less than 5	221	52.4
	Five and above 5	201	47.6
**Marital status**	Single	31	7.3
	Married	344	81.5
	Divorce	26	6.2
	Widowed	21	5.0
**Religion of household head**	Orthodox	264	62.6
	Protestant	52	12.3
	Muslim	106	25.1
**Main means of income**	Merchant	86	20.4
	Government employee	88	20.9
	Private employee	125	29.6
	Daily labour	112	26.5
	Urban Farming	2	.5
	Microenterprise	4	.9
	Support	5	1.2
**Educational level of household**	Illiterate	52	12.3
	Read and write	35	8.3
	Primary	102	24.2
	Secondary	155	36.7
	Diploma and above	78	18.5
**Cigarette smoking**	Yes	62	14.7
	No	360	85.3

Regarding the housing units, 224 (53.0%) of the kitchens were attached to the house and 198 (47.0%) were detached from the house. About 203 (48.0%) households cook in a room that is also used for living/sleeping while 97 (23.0%) households had separate rooms for cooking and sleeping and 25 (6.0%) households cook outside of the house. The means of ventilation used 125 (52.0%) households are windows and open doors. However, 115 (48.0%) households were found to have no proper ventilation.

### Association between the prevalence of acute respiratory infections and biomass fuel

About 145 households (34.4%) used charcoal, 89 households (21.1%) used wood fuel, 82 households (19.4%) used electricity, 76 households (18.0%) used kerosene, 18 households (4.3%) used urban residues and leaves, 10 households (2.4%) used LPG.

The prevalence of ARI observed during the two week period included 76 cases of ARI (29.9%) in households using biomass fuels (fuel wood, dung cakes, residues and/or charcoal), 16 cases (21.1%) in households using kerosene, and 9 cases (9.8%) in households using clean fuels. The overall prevalence of ARI in the sample was found to be 23.9%.

Bivariate logistic regressions were used to estimate the relative association of household energy use and other exposure variables on ARI. Children of female headed homes were 44% less likely to have ARI than males (COR 0.56; 95% CI: 0.35-0.89). Cigarette smoking was associated with ARI. Children living in a smoking family were 2 times more likely to have ARI than that of non-smokers (COR 1.96; 95% CI: 1.10-3.49) (Table 2). Mother’s behavior towards cooking places in relation to the child’s location was also analyzed. Children who were held by their mothers were nearly two times more likely to suffer from an ARI than children who were not held by their mothers while cooking (COR 1.88; 95% CI: 1.19- 2.95) (see Table 2).

**Table 2:** **Bivariate analysis of household characteristics on ARI prevalence in children in Addis Ababa, February 2012**

Variables	No. of children with ARI (n = 422)		Crude OR(95%CI)
	Yes	No	
**Sex of household head**			
Male	58	227	0.56(0.35, 0.89)
Female	43	94	1.0
Family size*			
less than five	54	167	1.0
Five and above	47	154	1.06(0.68, 1.66)
**Educational level**			
Illiterate	17	35	2.22(0.99, 5.03)
Read and write	10	25	1.83(0.72, 4.65)
Primary	24	78	1.40(0.67,2.94)
Secondary	36	119	1.38(0.70, 2.75)
Diploma and above*	14	64	1.0
**Cigarettes smoke**			
Yes	22	40	1.96(1.10,3.49)
No*	79	281	1.0
**Holding child at backduring cooking**			
Yes	51	113	1.88(1.19, 2.95)
No*	50	208	1.0

*reference category.

Children live in home without proper ventilation was two times more likely suffering from ARI than in ventilated home (OR 2.41; 95% CI: 1.34-4.32) (Table 3).

**Table 3:** **Bivariate analysis of housing& kitchen characteristics effects on ARI prevalence in children in Addis Ababa, February 2012**

Variables	No. of children with ARI (n = 422)		Crude OR(95%CI)
	Yes	No	
**House type**			
Made from mud	90	211	2.05(0.76, 5.54)
Brick, stone and block	6	86	0.34(0.10,1.19)
From both*	5	24	1.0
**House ventilation**			
Yes*	16	101	1.0
No	84	220	2.41(1.34,4.32)
**Kitchen characteristics**			
Living room*	52	151	1.0
Separate room	17	80	0.61(0.34,1.14)
Separate building	24	73	0.96(0.56,1.67)
Open air	8	17	1.37(0.56, 3.35)

*reference category.

The distribution of children ARI prevalence with respect to fuel and stove type characteristics is summarized in (Table 4). Overall, biomass, kerosene, and all stoves except improved biomass stoves are strongly associated with increased probability of ARI among children.

**Table 4:** **Bivariate analysis of effects of household fuels use and stove type on ARI prevalence in children in Addis Ababa, February 2012**

Variables	Children with ARI (n = 422)		Crude OR(95%CI)
	Yes	No	
**Fuel type**			
Biomass fuel	76	178	3.98(1.88, 8.24)
Kerosene	16	60	2.46(1.02, 5.94)
Clean fuels*	9	83	1.0
**Stove type**			
Traditional biomass stove	37	58	5.42(2.50, 11.76)
Improved biomass stove	6	28	1.82(0.61,5.46)
Kerosene stove	18	61	2.51(1.08, 5.81)
Charcoal stove	30	89	2.87(1.32, 6.23)
Clean stove*	10	85	1.0

*reference category.

### Multivariate logistics regression analysis of household energy use and other exposure variables on ARI

Variables that have a p-value less than 0.30 with ARI in a bivariate analysis were subjected to a multivariate logistics regression analysis. fuel use, sex of head of households, cigarette smoking, ventilation, and holding a child on the mothers back while cooking were Included in model (see Table 5).

**Table 5:** **Multivariate analysis effects of fuels used for cooking on ARI prevalence in children in Addis Ababa, February 2012**

Variables	No. of children with ARI (n = 422)		Crude OR(95% CI)	Adjusted OR(95% CI)
	Yes	No		
**Fuel type**				
Biomass fuel	76	178	3.98(1.88, 8.24)	2.96(1.38, 3.87)
Kerosene	16	60	2.46(1.02, 5.94)	1.96(0.78, 4.89)
Clean fuels*	9	83	1.0	1.0
**Sex**				
Male	58	227	0.56(0.35, 0.89)	0.59(0.36,0.96)
Female*	43	94	1.0	1.0
**Smoke**				
Yes	22	40	1.96(1.10,3.49)	2.05(1.11, 3.78)
No*	79	281	1.0	1.0
**Ventilation**				
Yes*	16	101	1.0	1.0
No	84	220	2.41(1.34,4.32)	2.09(1.13,3.87)
**Child holding during cooking**				
Yes	51	113	1.88(1.19, 2.95)	1.78(1.09, 2.87)
No*	50	208	1.0	1.0

*reference category.

The odds of ARI was 2.96 (95% CI: 1.38-3.87) in households using biomass fuel relative to households using cleaner fuels after adjusting for sex, smoking, ventilation, child handling behavior. Overall, type of fuel, sex of the household, ventilation, and child handling behavior were important predictors of ARI among under- five children.

## Discussion

Of the study households, biomass fuel (charcoal, fuel wood, residues and dung) was the most widely used fuel for cooking in Addis Ababa. Fewer households use electricity and LPG as cooking fuel, approximately one-fifth of the households sampled.

The result of this study indicated two weeks prevalence of ARI among children under-five years was 23.9%. Nearly, one-fourth of children that are highly vulnerable population groups living in slum area of Addis Ababa were suffering from both upper and lower respiratory infection. This Study was comparable with study conducted in shebadeno southern Ethiopia 21% [12] and some studies in conducted in Africa , particularly well comparable with ARI prevalence of Ethiopia (24%) in 2000 [13], Study conducted in Tanzania ARI in the sample was found to be 29.76% prevalence for children under age 5 in Tanzania [14]. But slightly lower than a study in Zimbabawe shows 16% children suffered from ARI [15] and Ethiopia national figure prevalence of (13%) in 2005 [16]. This might be because children in the inner slum city stay in homes being exposed to indoor air pollution from biomass fuel due to space scarcity to stay outside. Underlying risk factors such as malnutrition among children may also aggravate the problems.

The sex of the head of household was significantly associated with ARI prevalence in children. This shows that children who live in male- headed homes less exposed to ARI than female- headed. This result is contrary to existing literature and expected results. A previous study conducted in Ethiopia indicated no significant association of ARI prevalence with sex of household head [12]. A study in urban household in Nigeria also indicated that the sex of household heads did not show any relationship between with the use of biomass fuel use [17]. This inconsistency may be due to socio-economic factors.

Cigarette smoking is also associated with ARI prevalence. Children live in a smoking household are two times more likely to suffer from ARI than that of non-smoking households. This may increase the concentration of indoor air pollution concentration which aggravate ARI in children. This was consistent with the studies conducted in Africa, in Kenya smoking increased the risk of ARI with an odds ratio of 1.48 (95% CI 1 · 07–2.04) in the logistics model [2]. Another study also showed that cigarettes smoking increased the risk of ARI from airborne pollutants in young children [17]. Similarly, a Study conducted in an urban slum of India indicated that smoking habits strongly influences the incidence of ARI [18].

This study shows strong associations with increased between biomass fuel and prevalence of ARI in children. Children living in households that are reliant on biomass fuels are about three times more likely to suffer from an ARI compared to children living in homes reliant on clean fuels. This implies that the children exposure-response for biomass fuel smoke is the most toxic among all the fuel types. This was followed by kerosene and electricity/natural gas. This result similar with most studies conducted in Africa. Study in Zimbabwe showed that children in households using wood, dung, or straw for cooking were more than twice as likely to suffer from an ARI compared to children from households using LPG or electricity [15].

This study was consistent with study in South Africa on children living in households that are reliant on solid fuel are 2 to 4 times more likely to suffer from an Acute Lower Respiratory Infection (ALRI) compared to children living in homes reliant on electricity [19]. But inconsistence with study in Tanzania indicated that the effect of biomass fuel on ARI is the same as the effect of kerosene [20]. A Nigerian study also showed that the use of unclean energy among urban households is confounded with health related problems of IAP [21].

Children who live in households without ventilation were about two times more likely to suffer from an ARI compared to children who live in than in ventilated households. Study indicated child holding at back during cooking is associated with children ARI prevalence. Children in home mother hold child at back while cooking is about two times suffer from ARI). Patterns of time-activity, which place children near sources of pollution such as cooking stoves, contribute to the increased risk of ARI from airborne pollutants in young children [22].

This study provided quantitative snapshot of urban homes using a variety of fuels. Households in this study were grouped based on whether there were children living in the homes and type of cooking fuel used (wood, residues, charcoal, kerosene LPG or electricity). But it is common in Addis Ababa to find one house using more than one fuel. Such grouping home without more information on children from homes using a combination of several fuels may lead to different effect values on children ARI prevalence. ARI was assessed by the use questionnaire which required mothers to explain on the health of their children with respect to ARI in the period two weeks prior to the survey. Since this is not an objective way of collecting health information, the method used to collect health information may have caused underestimation or overestimation of ARI frequency in children.

## Conclusion

Biomass fuel is the dominant fuel used for cooking in Addis Ababa, Ethiopia. This study shows strong associations between biomass fuel and prevalence of ARI in children under-five year. Apart from family size, educational level and kitchen characteristics others factors like sex of head of house hold, cigarette smoking habits, fuel type, ventilation , holding child at back while cooking a influence the prevalence of ARI in children under-five. Therefore, an effective mitigation strategy should employ multiple interventions such as improvements in fuels, cooking technologies, ventilation conditions. Additional behavior adjustments such as ensuring children are kept away from smoke and not smoking in closed range of children. The relationship needs to be further investigated using more direct measures of IAP exposure and clinical measures of acute respiratory infection needed to confirm the association. Other cofounders like nutritional status, immunization status, and behavior should also be studied.
